# Transcriptomic predictors of prostate cancer recurrence following focal cryotherapy: a pooled analysis of phase II trial and prospective cohort data

**DOI:** 10.1016/j.jncc.2025.04.002

**Published:** 2025-05-29

**Authors:** Kae Jack Tay, Boon Hao Hong, Enya Hui Wen Ong, Kah Min Tan, Gianella Cabuhat Pacho, Samantha Jingxuan Wong, Yu Guang Tan, Yan Mee Law, Nye Thane Ngo, Puay Hoon Tan, John S.P. Yuen, Henry S.S. Ho, Kenneth Chen, Jiping Peng, Clare Wei Tian Foo, Xin Xiu Sam, Jeffrey K.L. Tuan, Ravindran Kanesvaran, Rajan T. Gupta, Steven Rozen, Thomas J. Polascik, Yang Liu, James Proudfoot, Elai Davicioni, Li Yan Khor, Melvin Lee Kiang Chua

**Affiliations:** 1 Department of Urology, Singapore General Hospital, Singapore; 2 Duke-NUS Medical School, Singapore; 3 Division of Medical Sciences, National Cancer Centre Singapore, Singapore; 4 Department of Diagnostic Radiology, Singapore General Hospital, Singapore; 5 Division of Pathology, Singapore General Hospital, Singapore; 6 Luma Medical Clinic, Singapore; 7 Department of Anatomical Pathology, Singapore General Hospital, Singapore; 8 Division of Radiation Oncology National Cancer Centre Singapore, Singapore; 9 Division of Medical Oncology National Cancer Centre Singapore, Singapore; 10 Department of Radiology, Duke Cancer Centre, Durham, USA; 11 Department of Biostatistics and Bioinformatics, Duke University School of Medicine, Durham, USA; 12 Department of Urology, Duke Cancer Centre, Durham, USA; 13 Veracyte. Inc, San Diego, USA

**Keywords:** Focal therapy, Cryotherapy, Prostate cancer, Decipher genomic classifier

## Abstract

**Objective:**

Focal therapy (FT) is a potential treatment option for limited-volume clinically-significant prostate cancer (csPCa). However, despite rigorous selection, approximately 20% of patients experience early failure. We investigated the association of transcriptomic profiles and csPCa recurrence post-FT.

**Methods:**

52 men from a phase II trial (NCT04138914) and a prospective observational cohort underwent focal cryotherapy for csPCa. Patients underwent multiparametric magnetic resonance imaging, and targeted and systematic-saturation biopsy before- and 1-year post-FT. Recurrence was defined as grade-group (GG) ≥2 cancer in the 1-year post-FT biopsy. Pre-treatment lesions were profiled using the Decipher genomic classifier (GC). GC scores, luminal-basal status, tumor microenvironment and cancer hallmark pathways were correlated with csPCa recurrence.

**Results:**

Median PSA was 7.0 ng/dl; 37/52 (71.1%) men had GG2, 12/52 (23.1%) GG3, and 3/52 (5.8%) GG4 cancer. Recurrence was observed in 9/52 (17.3%) men. Median GC score was higher in patients with recurrence (0.60 *vs* 0.38, *P* = 0.014) and remained significantly associated with recurrence after adjustment for GG (adjusted *OR*: 1.37 [95% CI: 1.01–1.93], *P* = 0.04). Luminal-proliferative tumors based on the prostate cancer-specific subtyping classifier (PSC) had more csPCa recurrence compared with luminal-differentiated (LD) and basal subtypes (30.4% *vs* 0% [LD] *vs* 15.4% [basal-neuroendocrine] and 14.3% [basal-immune], *P* = 0.027). Higher expression of DNA repair pathway was also associated with recurrence (*OR*: 2.12 [95% CI: 1.09–4.57], *P* = 0.025).

**Conclusions:**

Higher GC score is associated with risk of csPCa recurrence post-FT. Patients with GC low-risk and PSC-LD csPCa may represent the ideal subgroup for FT. Prospective validation in a large cohort is warranted.

## 1 Introduction

Focal therapy (FT), the targeted destruction of only the cancerous area within the prostate, is a potential treatment option for limited-volume clinically significant prostate cancer (csPCa).^1^ If csPCa foci can be ablated in their entirety, adequate oncological control could be obtained while sparing adjacent anatomical structures thus preserving quality of life.

To do this, csPCa lesions must be accurately identified and defined through imaging and biopsy to determine the appropriate location and volume of ablation. Post-treatment, imaging and biopsy is equally important in determining the recurrence or persistence of csPCa inside (infield) or outside the treated area (outfield).^2^ While awaiting long-term data, imaging and re-biopsy may provide the next best surrogate marker of early oncological control.^3^

However, despite rigorous selection, some patients will fail FT. Studies that have mandated re-biopsy of the prostate within a year of FT report average overall csPCa recurrence rates of approximately 20%.^4^ Similarly, despite utilizing magnetic resonance imaging (mpMRI) followed by targeted and extensive mapping prostate biopsies to select patients for FT in a phase II trial of focal cryotherapy for csPCa, we observed a 21.8% csPCa recurrence rate 1-year post-FT.^5^ This rate of recurrence is similar to that reported in other focal therapy series^6^ and mirrors the findings of studies correlating whole-mount histology at radical prostatectomy following MRI- transrectal ultrasound (MRI-TRUS) fusion biopsy.^7^

There is a need to better stratify limited-volume csPCa patients to FT *versus* upfront radical therapy. Beyond the standard clinical parameters, there is currently no evidence supporting the use of any molecular biomarker to select patients for FT.^8^ Here, in an extended prospective cohort of a clinical trial undergoing mandatory repeat imaging and biopsy, we report our findings in interrogating whether a pre-treatment genomic classifier (GC) is associated with csPCa recurrence 1-year post-FT.

## 2 Materials and methods

### 2.1 Patient selection and ethical approval

Patients underwent FT using focal cryotherapy from 2019 to 2021 in a single-arm Phase II prospective clinical trial (NCT04138914) and subsequently, in a prospective observational (PO) cohort extension from 2021 to 2023 utilizing identical patient selection, treatment and follow-up protocols (Supplementary methods), the only difference being that FT was grant-sponsored in the trial cohort and patient-sponsored in the PO cohort. FT was offered to patients with localized prostate cancer (PCa), no prior active PCa treatment, prostate-specific antigen (PSA) < 20 ng/ml, biopsy prognostic grade-group (GG) ≤ 4, and csPCa lesion volume of ≤ 3 ml for single lesions or ≤ 1.5 ml each for two lesions.^8^^,^^9^

### 2.2 Pre-treatment lesion identification

All patients underwent 3-Tesla mpMRI of the prostate (Magnetom Skyra, Siemens Healthineers, Erlangen, Germany) using a 60-channel pelvic phase array coil. mpMRI was performed using our MRI (PIRADS)v2.1 compliant standard institutional protocol previously described^10^ (details in Supplementary methods), and interpreted by experienced uro-radiologists with experience in reading prostate mpMRI. Each lesion was graded and assigned a PIRADSv2.1 category.

Robotic transperineal MRI-TRUS fusion targeted biopsy was performed for PIRADS ≥ 3 lesions along with a non-targeted systematic saturation prostate biopsy of at least 20 cores.^11^ The mpMRI and biopsy outcomes were correlated by a multidisciplinary team, comprising of a radiologist (Y.M.L.), pathologist (N.N.T.) and urologist (K.J.T.) for eligibility.

### 2.3 Lesion gene expression profiling

**Fig. 1:**
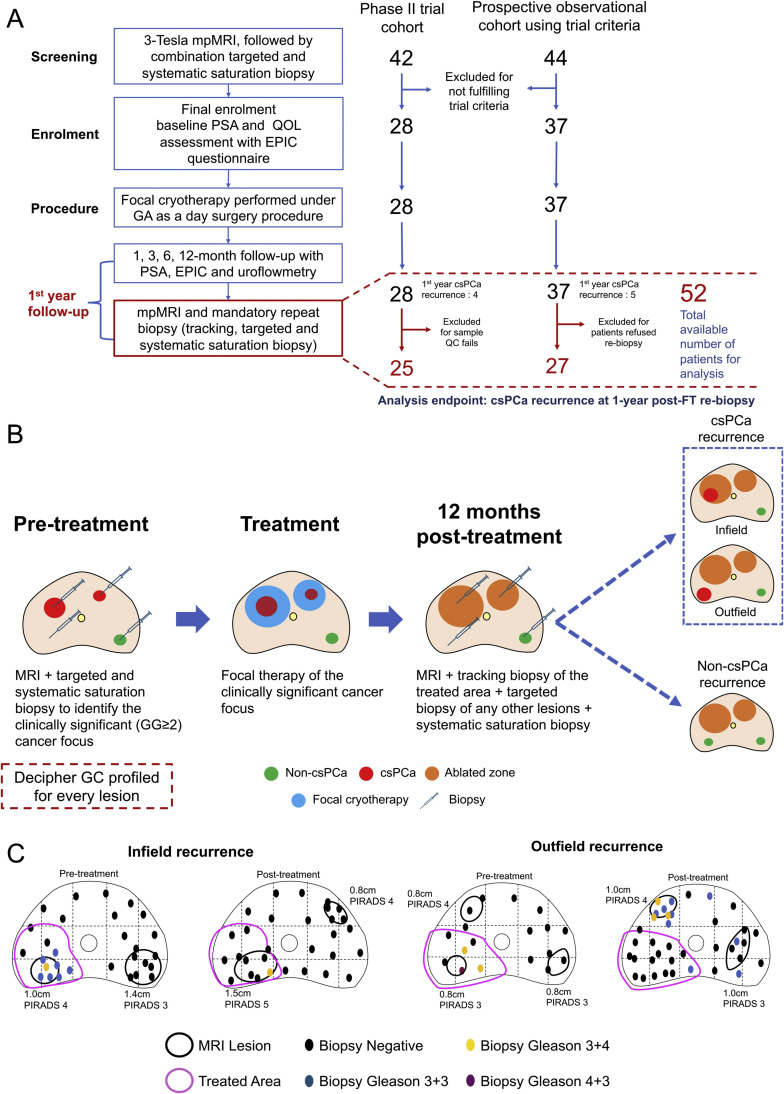
(A) CONSORT diagram of study consisted of prospective phase II trial cohort and prospective observational cohort using trial criteria. (B) Overview of biopsy and tumor sampling schema in pre-treatment. (C) The selected case to illustrate the definition of infield and outfield csPCa recurrence of a patient who underwent focal cryotherapy. CsPCa, clinically significant prostate cancer; EPIC, European Prospective Investigation into Cancer; FT, focal therapy; GA, general anesthesia; GC, genomic classifier; GG, Gleason grade; mpMRI, magnetic resonance imaging; QC, quality control; QOL, quality of life; PSA, prostate-specific antigen.

Formalin-fixed paraffin-embedded (FFPE) diagnostic biopsies were obtained for each lesion identified as cancer on mpMRI/ biopsy (Fig. 1B). An expert genitourinary pathologist (L.Y.K.) delineated the tumor region bearing the highest GG with a minimum cellularity of 70%. Sample processing for gene expression profiling using the Decipher assay (Veracyte, San Diego, CA) was performed as previously described^12^ (Supplementary methods - Gene expression profiling).

### 2.4 Focal cryotherapy

Our focal cryotherapy (Visual ICE™, Boston Scientific, Boston, USA) technique was previously described^13^ (Supplementary methods). Two freeze-thaw cycles were performed under real-time ultrasound guidance to achieve a 5 mm treatment margin of < 20 degrees Celsius around the target lesion/zone.^10^ A maximum of 2 lesions were treated.

### 2.5 Post-treatment evaluation of the treated and untreated areas

1-year post-treatment, patients underwent a mandatory repeat prostate mpMRI and biopsy, comprising: (1) targeted biopsy of the previous ablation sites, (2) targeted biopsy of any new PIRADS category ≥ 3 lesions and (3) systematic saturation biopsy of the remaining prostate.

### 2.6 Outcomes and analysis

The primary clinical outcome was detecting any csPCa (GG ≥ 2) at 1-year follow-up via mpMRI and targeted/systematic biopsy, further stratified into infield and outfield csPCa recurrence (Fig. 1C). The detailed analysis plan can be found in the Supplementary methods - Exploratory data analyses. Briefly, the association of the GC score to csPCa recurrence at 1-year repeat biopsy was assessed by comparing it to GG and PIRADS categories. For patients with multifocal cancer, the highest GC score, GG, and PIRADS category were used as representative data points. To explore the transcriptomic differences in limited-volume csPCa tumors, lesion-level analyses were performed by including all profiled lesions for each patient, thereby increasing the statistical power of the analyses. The transcriptomic signatures such as luminal-basal status (prostate cancer subtyping classifier [PSC], and Prediction Analysis of Microarray 50 [PAM50]),^14^^,^^15^ immune cell signatures,^16^ and cancer hallmark pathway^17^ were downloaded from the Genomics Resource for Intelligence Discovery database and used for the analyses. Rv4.2.2 was used in all statistical analyses.

## 3 Results

### 3.1 Patient characteristics of trial and PO cohorts

We recruited 65 patients from March 2019 to September 2022 for this study: 28 from a phase II trial (March 2019 to January 2021) and 37 from the PO cohort extension (February 2021 to September 2022). Of these patients, 52 were eligible for analyses (10 from PO cohort excluded for refusing 1-year repeat biopsy, and 3 from trial cohort contributed specimens that failed quality control, Fig. 1A). Patient demographics of both cohorts are summarized in Table 1. Overall, clinical parameters were comparable between them, except for the lower median age in the PO than the trial cohort (71.5 [IQR: 64.8–74.2] years *vs* 65.9 [IQR: 61.2–71.1] years, *P* = 0.012). Notably, the PO cohort did not have any patients with GG4 PCa at the time of reporting.

**Table 1:** Patient demographics characteristics.

Characteristics of patients	Pooled (*n* = 52)	Trial (*n* = 25)	PO (*n* = 27)	*P* value (Trial *vs* PO)
Age at diagnosis, median (IQR), years	69.0 (62.8–73.0)	71.5 (64.8–74.2)	65.9 (61.2–71.1)	0.012
PSA at diagnosis, median (IQR), ng/dl	7.0 (6.0–9.1)	7.2 (6.2–9.6)	7.0 (5.0–8.8)	0.395
Grade group, No. (%)				
2	37 (71.1)	18 (72.0)	19 (70.4)	0.117
3	12 (23.1)	4 (16.0)	8 (29.6)	
4	3 (5.8)	3 (12.0)	–	
NCCN risk group, No. (%)				
FavInt	31 (59.6)	13 (52.0)	18 (66.7)	0.155
UnFavInt	18 (34.6)	9 (36.0)	9 (33.3)	
High	3 (5.8)	3 (12.0)	–	
GC score, median (IQR)	0.44 (0.24–0.64)	0.45 (0.25 – 0.67)	0.41 (0.22–0.62)	0.819
csPCa recurrence, No. (%)				
Yes	9 (52.8)	4 (16.0)	5 (18.5)	0.457
No	8 (15.4)	5 (20.0)	3 (11.1)	
Number of lesions per patients, No. (%)				
1	27 (51.9)	13 (52.0)	14 (51.9)	0.712
2	19 (36.6)	10 (40.0)	9 (33.3)	
3	6 (11.5)	2 (8.0)	4 (14.8)	

Abbreviations: CsPCa, clinically-significant prostate cancer; GC, genomic classifier; IQR, interquartile range; MRI, magnetic resonance imaging; NCCN, National Comprehensive Cancer Network; PO, prospective observational.

Fig. 1B illustrates the specimen collection schema of patients from both cohorts. We collected and analyzed a total of 83 histologically-confirmed lesions (39 from the trial cohort and 44 from the PO cohort) from 52 patients (Table 2); 27/52 (51.9%) patients had 1 lesion, 19/52 (36.6%) had 2 lesions, and 6/52 (11.5%) had 3 lesions. Of the 83 lesions, 61 (73.5%) were MRI visible (13 were classified as PIRADS-3, 34 as PIRADS-4, and 14 as PIRADS-5), while 22 (26.5%) were MRI invisible. By GG, 22/83 (26.5%) lesions were GG1, 46/83 (55.4%) were GG2, 12/83 (14.5%) were GG3, and 3/83 (3.6%) were GG4. Location-wise within the prostate, 41/83 (49.4%) lesions were anterior and 42/83 (50.6%) posterior.

**Table 2:** Patients’ profiled lesions characteristics.

Characteristics of lesions	Overall (*n* = 83)	Trial (*n* = 39)	PO (*n* = 44)	*P* value (Trial *vs* PO)
Grade group, No. (%)				
1	22 (26.5)	9 (23.0)	13 (29.5)	0.189
2	46 (55.4)	23 (59.0)	23 (52.3)	
3	12 (14.5)	4 (10.3)	8 (18.2)	
4	3 (3.6)	3 (7.7)	0	
PIRADS, No. (%)				
MRI invisible	22 (26.5)	13 (33.3)	9 (20.5)	0.348
3	13 (15.7)	5 (12.9)	8 (18.2)	
4	34 (41.0)	13 (33.3)	21 (47.7)	
5	14 (16.8)	8 (20.5)	6 (13.6)	
Biopsy cores obtained, median (IQR)	6 (3–9)	7 (3–9)	6 (4–9)	0.855
Biopsy cores positive for any cancer, median (IQR)	2 (1–5)	3 (1–6)	2 (2–4)	0.893
Biopsy cores positive for CS cancer, median (IQR)	1 (0–3)	1 (0–4)	1 (0–2)	0.297
Highest % core involvement, median (IQR)	45.0 (17.5–67.5)	40.0 (10.0–60.0)	45.0 (23.8–70.0)	0.580
Location, No. (%)				
Anterior	41 (49.4)	20 (51.3)	21 (47.7)	0.918
Posterior	42 (50.6)	19 (48.7)	23 (52.3)	
Treated, No. (%)	72 (84.7)	36 (92.3)	36 (81.8)	0.239
GC score, median (IQR)	0.3 (0.2–0.5)	0.3 (0.2 – 0.5)	0.3 (0.2 – 0.5)	0.675
Infield recurrence, No. (%)	7 (8.3)	5 (12.5)	2 (4.5)	0.313

Abbreviations: CS, clinically-significant; GC, genomic classifier; IQR, interquartile range; MRI, magnetic resonance imaging; PIRADS, Prostate-Imaging Reporting and Data System; PO, prospective observational.

Of the 83 lesions, 72 (84.7%) were treated with FT; of which, 61 were csPCa lesions, while the remaining 11 were non-csPCa lesions that were treated because either they were near a csPCa lesion (*n* = 9), or treatment was performed at the patients’ request (*n* = 2). Altogether, we recorded 9/52 (17.3%) csPCa and 8/52 (15.4%) non-csPCa recurrences based on the outcomes of 1-year post-FT repeat saturation and MRI-targeted biopsy (Fig. 1A). Of the patients with csPCa recurrences, 3/9 (33.3%) were infield only, 4/9 (44.4%) were outfield only, and 2/9 (22.2%) were recorded as synchronous infield and outfield recurrences.

### 3.2 Correlation of radiological, pathological, and transcriptomic profiles by lesion

**Fig. 2:**
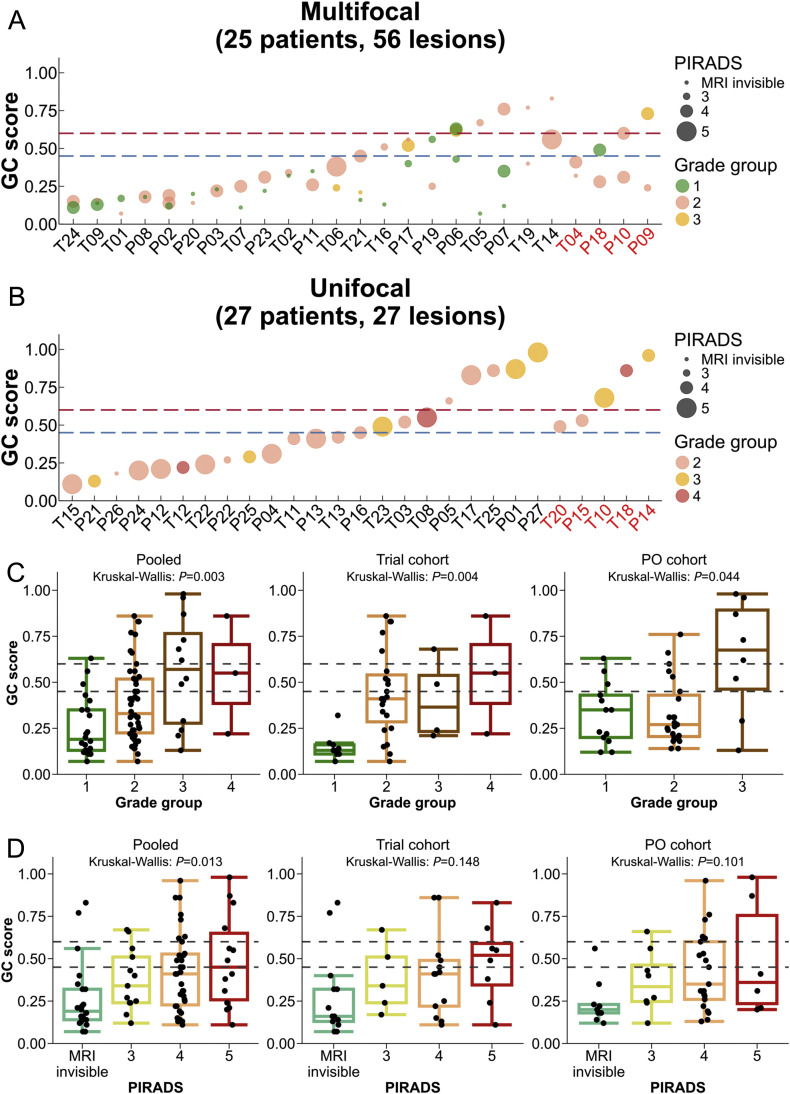
Intra-patient variation of Decipher GC scores relative to the Gleason grade group and PIRADS categories for each lesion sampled from PCa patient. (A and B) Intra-patient variation of multifocal PCa patients (A) and unifocal PCa patients (B). The x-axis in (A) and (B) represents patient IDs, where patients coloured in red indicate csPCa recurrence 1-year post-FT, and those in black indicate non-csPCa recurrence 1-year post-FT. (C and D) Correlation between GC scores with selected variables in pooled cohort from phase II trial and PO cohorts classified by Gleason grade groups (C) and PIRADS categories (D). CsPCa, clinically significant prostate cancer; GC, genomic classifier; MRI, magnetic resonance imaging; PCa, prostate cancer; PIRADS, Prostate-Imaging Reporting and Data System; PO, prospective observational.

We first investigated the intra-patient variation of GC scores for the 25 patients with multifocal PCa, relative to the GG and PIRADS category of each lesion sampled (Fig. 2A). GC risk groups were concordant across all lesions in 14/25 (56.0%) patients, while 11/25 (44.0%) patients had lesions that spanned multiple GC risk-groups primarily between low (GC < 0.45) and intermediate (GC > 0.45). In 23/25 (92%) the highest GC score was observed in the lesion with highest GG. The two patients with discordant GC and GG are detailed in Supplementary Table 1.

Overall, we observed that GC scores were correlated with GG (Spearman *R* = 0.41, *P* = 0.0001, Fig. 2A-C), with a consistent trend between the trial (*R* = 0.52, *P* = 0.0006) and PO cohorts (*R* = 0.29, *P* = 0.057; Fig. 2C). Similarly, we observed a trend between GC scores and PIRADS categories in the pooled analysis (*R* = 0.34, *P* = 0.013, Fig. 2D), although these trends did not reach statistical significance in the individual cohorts (trial: *P* = 0.148, RW: *P* = 0.101). Nonetheless, we observed large ranges of GC scores within each GG and PIRADS category.

### 3.3 GC scores were associated with csPCa recurrences following FT

**Fig. 3:**
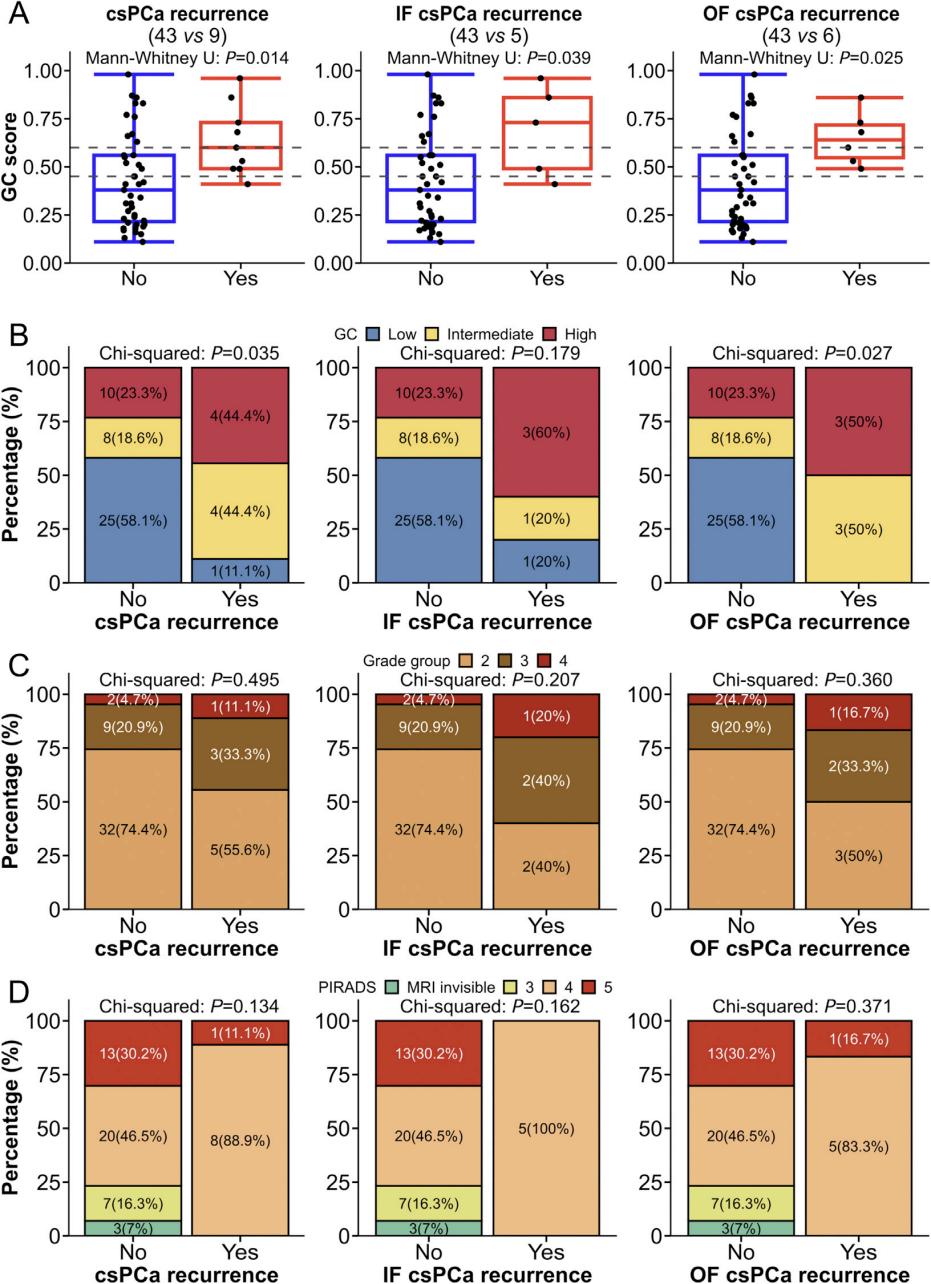
(A-D) The association of overall, infield and outfield csPCa recurrences detection 1-year post-focal therapy with radiological, pathological, and transcriptomic profiles classified by GC scores (A), GC classes based on previous established cut-off points (B), GG groups (C), PIRADS categories (D). CsPCa, clinically significant prostate cancer; GC, genomic classifier; IF, infield; MRI, magnetic resonance imaging; OF, outfield; PIRADS, Prostate-Imaging Reporting and Data System.

Next, we tested the association between GC scores, GG, and PIRADS category with the occurrence of csPCa at 1-year post-FT. For patients harboring multifocal PCa, the highest GC score, GG, and PIRADS category was used as the representative datapoint of that subject for this analysis. Of the 3 parameters, only GC score was associated with csPCA recurrence (Table 3). GC scores were significantly higher in the 9 patients who manifested csPCa recurrences at 1-year post-FT compared with 43 patients who did not recur or had non-csPCa recurrences (median 0.60 *vs* 0.38, *P* = 0.014, Fig. 3A), irrespective of whether the recurrence occurred infield (*P* = 0.039) or outfield (*P* = 0.025). A similar trend was observed when stratified by GC risk-groups; 8/9 (88.8%) patients with csPCa recurrence were classified as GC high- and intermediate-risk in contrast to 18/43 (41.9%) patients without csPCa recurrence (Chi-squared *P* = 0.035). Notably, only 1 (4.0%) of 25 patients who was stratified as GC low-risk experienced a csPCa recurrence post-FT.

**Table 3:** Univariable logistic regression analyses between selected variables and csPCa recurrence.^a^

	Univariable		Multivariable	
	Odd ratio (95% CI)	*P* value	Odd ratio (95% CI)	*P* value
Age at diagnosis	0.97 (0.88-1.08)	0.609		
PSA at diagnosis	1.18 (0.93-1.50)	0.168		
Focality				
Unifocal	Reference			
Multifocal	0.86 (0.20-3.43)	0.825		
MRI tumour size	0.74 (0.19-2.70)	0.650		
PIRADS				
< 5	Reference			
5	0.39 (0.04-1.99)	0.276		
Grade group				
2	Reference			
3	2.18 (0.44-9.92)	0.326	1.23 (0.20-6.28)	0.812
4	3.55 (0.29-32.56)	0.288	2.40 (0.16-26.44)	0.491
NCCN risk group				
FavInt	Reference			
UnFavInt	3.32 (0.77-16.26)	0.109		
High	4.89 (0.37-50.39)	0.202		
GC score	1.41 (1.06-1.98)	0.019	1.37 (1.01-1.93)	0.040

a Multivariable logistics regression analysis between csPCa recurrence and GC score by adjusted with grade group. The number of patients include: csPCa recurrence, yes (*n* = 9); no (*n* = 43). Abbreviations: CI, confidence interval; CsPCa, clinically-significant prostate cancer; GC, genomic classifier; MRI, magnetic resonance imaging; NCCN, National Comprehensive Cancer Network; PSA, prostate-specific antigen; PIRADS, Prostate-Imaging Reporting and Data System.

We did not observe an association for GG and PIRADS category with csPCa recurrence in our cohort (Fig. 3C and D). Univariable and multivariable analyses of association between the clinical and genomic parameters and odds of csPCa recurrence indicated that continuous GC score was the only significant predictor of event, even when adjusted for GG (Adjusted*OR*: 1.37 [95% CI: 1.01–1.93], *P* = 0.04, Table 3) and PIRADS (Adjusted*OR*: 1.56 [95% CI: 1.13–2.37], *P* = 0.006, Supplementary Table 2).

### 3.4 Correlation of luminal-basal subtype and tumor microenvironment signatures with csPCa following FT

Given the significant association of GC with csPCa recurrence following FT, we sought to interrogate if the somatic tumor and microenvironment profiles were associated with post-FT failures. To this end, we explored individual lesion luminal-basal subtypes,^14^^,^^15^ and immune signatures,^16^ cancer hallmarks,^17^ based on previous work indicating the relevance of these profiles in East Asian men with PCa,^12^ of all 83 profiled lesions and tested their associations with the recurrence of csPCa.

**Fig. 4:**
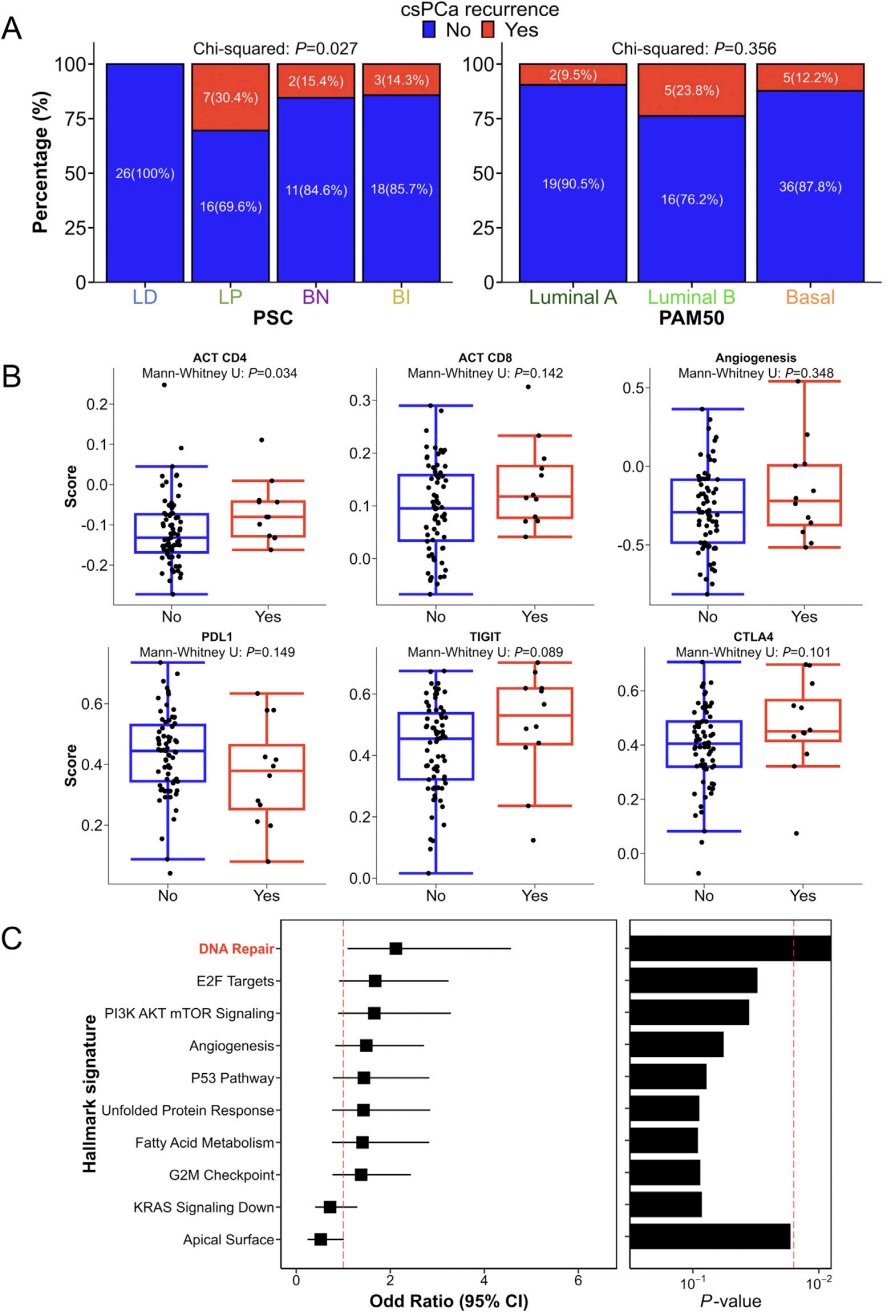
The association of luminal-basal status, tumor immune microenvironment signatures with csPCa recurrences related lesions in 1-year post-focal therapy. (A) PSC and PAM50. (B) Selected immune signatures and angiogenesis. (C) Top-10 prostate cancer related hallmark signatures based on *P*-value. ACT, adoptive cell transfer therapy; BI, basal-immune; BN, basal-neuroendocrine; CsPCa, clinically significant prostate cancer; LD, luminal-differentiated; LP, luminal-proliferative; PAM50, prediction analysis of microarray 50; PSC, Prostate-specific subtyping classifier.

We noted a statistically significant difference in csPCa recurrence rates between molecular subtypes (Fig. 4A). Notably, lesions of the PSC luminal-differentiated (LD) subtype demonstrated 0/26 (0%) recurrences, luminal-proliferative (LP) 7/23 (30.4%), basal-neuroendocrine (BN) 2/13 (15.4%), and basal-immune (BI) 3/21 (14.3%) recurrences respectively (*P* = 0.027). PSC-LD lesions were less likely associated with csPCa recurrence by compared to other PSC subtypes even after adjustment for GG (Adjusted*OR*: 0.09 [95% CI: 0.00–0.69], *P* = 0.015; Supplementary Table 3). PAM50 subtypes, on the contrary, were not associated with csPCa recurrences (*P* = 0.356).

Exploring the TIME signatures, we observed higher median levels of adoptive cell transfer therapy (ACT) CD4 expression (−0.08 *vs* −0.13, *P* = 0.034) in lesions associated with csPCa recurrence than those without (Fig. 4B). We did not observe significant differences in CTLA4, TIGIT, PDL1, ACT CD8 or angiogenesis between the two groups.

Lastly, we explored the association of 38 cancer hallmark pathways with csPCa recurrence; Fig. 4C illustrates the top 10 pathways based on their *P*-value. A higher DNA repair pathway score appeared associated with csPCa recurrence (Adjusted*OR*: 1.94 [95% CI: 0.97–4.20], *P* = 0.061; Supplementary Table 4).

## 4 Discussion

In men with localized PCa, standard-of-care remains radical surgery or radiotherapy. However, these definitive treatments are associated with substantial toxicities, and hence, active surveillance (AS) is considered in some patients.^18^^,^^19^ Between AS and definitive treatment, FT is an emerging treatment option for carefully selected patients with limited-volume csPCa. Nonetheless, despite advances in functional MRI and targeted biopsy, approximately 20% of men still develop csPCa recurrence within a year post-FT.^4^^,^^6^ This highlights the need to identify patients who fail very soon after FT, and should have been recommended for definitive treatment. Here, we conducted a correlative study in 52 patients who underwent comprehensive radiological workup and histological sampling as part of the study protocol prior to FT for their csPCa. Notably, pre-FT, all lesions were diagnosed using 3-Tesla mpMRI and transperineal targeted and systematic biopsy. The transperineal approach allowed us to sample the anterior and posterior parts of the prostate safely, at an equally high intensity.^20^, ^21^, ^22^ Indeed, the lesions we identified were distributed equally anteriorly (49.4%) and posteriorly (50.6%). In combination, each lesion was sampled with a mean of 6.6 biopsy cores. Post-FT, we found a 1-year csPCa recurrence rate of 17.3%, similar to that reported in international FT series using well-established modalities (cryotherapy, high-intensity focused ultrasound, and irreversible electroporation).^23^

We tested the hypothesis that Decipher GC outperforms conventional indices like PSA and GG in predicting csPCa recurrence at 1-year post-FT. We found that GC scores were significantly higher in the 9 patients who recurred compared with the rest of the cohort who either had a normal biopsy (*n* = 35) or biopsy harboring non-csPCa (*n* = 8) at 1-year post-FT (median score of 0.60 *vs* 0.38 *vs* 0.38). This trend was consistent independent of the site of recurrence. Additionally, GC score, as a continuous variable, was the only factor that was significantly associated with the likelihood of a positive 1-year post-FT biopsy (Adjusted *OR*: 1.37) after accounting for GG.

Using the established risk-categories for the GC, 26/52 (50.0%), 12/52 (23.1%), and 14/52 (26.9%) patients were defined as low-, intermediate-, and high-risk, respectively.^24^^,^^25^ Notably, only 1/26 (3.8%) GC low-risk patient developed a csPCa recurrence at 1-year post-FT, which is suggestive of a high specificity (96.2%) of GC low-risk for disease control.

To explore potential biological mechanisms beyond GC that underpin csPCa recurrence post-FT, we investigated the correlation of luminal-basal status and immune environment and cancer hallmark signatures with recurrence in our cohort. Our group had previously reported on the distinct luminal and basal PCa phenotypes that are characterized by dependency on the androgen receptor (AR) pathway, presence of oncogenic, and immune and neuroendocrine features.^12^ Then, among the different luminal-basal subtypes, PAM50-Luminal B and PSC-LP harbored the worst prognosis. It was thus interesting that, here, we also observed that lesions of the PSC-LP subtype were associated with the highest rate of csPCa recurrence, suggesting that this subgroup of patients is either not ideal for FT and deserve treatment intensification, or require closer surveillance post-FT.

Our study has several limitations. First, our findings are based on a limited cohort size and a low event rate, and thus only GG was used to adjust for GC, the main variable of interest. Exploratory analyses of cancer hallmark pathways and TIME were also not adjusted for multiple hypothesis testing. Second, standard cut-offs for Decipher GC may be suboptimal in a patient cohort with limited-volume csPCa undergoing FT. New thresholds will need to be established with larger cohorts. Third, we used mpMRI and comprehensive biopsy for patient selection. While this is current, there is increasing interest in using whole-body radionuclide imaging, such as prostate-specific membrane antigen positron emission tomography imaging for prostate cancer localization.^26^ Last, observations of recurrence were determined at 1-year and not beyond; some of these may represent technical ablative or selection failures rather than worse cancer biology.

Overall, our findings indicate that GC and luminal-basal subtyping has potential in predicting 1-year biopsy-based outcomes after FT but assessing it as a predictive biomarker will require more robust analysis and prospective validation. Nonetheless, this is the first correlative study of molecular predictors for FT outcomes conducted in a prospectively-enrolled cohort with strict eligibility criteria and surveillance protocol. These findings are a first step beyond standard clinical parameters in addressing a key obstacle in the adoption of FT as a mainstream strategy: Who is the best candidate for FT?

## 5 Conclusions

A transcriptomic genomic classifier is associated with the risk of csPCa recurrence 1-year post-FT independent of Gleason grade-group. Patients with GC low-risk and PSC-LD csPCa may represent the ideal subgroup for FT, whereas those with GC intermediate- or high-risk may be more suited to radical treatment or be more closely monitored if they elect for FT.

## Declaration of competing interest

Kae Jack Tay reports personal fees and non-financial support from Boston Scientific. Melvin L.K. Chua reports personal fees from Astellas, Pfizer, MSD, AstraZeneca, Varian, Janssen, IQVIA, Telix Pharmaceuticals; non-financial support from AstraZeneca; non-financial support from Veracyte Inc; grants from Ferring; personal fees and grants from Bayer; personal fees and grants from BeiGene; consults for immunoSCAPE Inc.; and is a co-inventor of the patent of a High Sensitivity Lateral Flow Immunoassay For Detection of Analyte in Sample (10202107837T), Singapore, and serves on the Board of Directors of Digital Life Line Pte Ltd that owns the licensing agreement of the patent, outside the submitted work. Ravindran Kanesvaran received personal fees from Astella, BMS, Ipsen, J&J, Merck, Novartis, Amgen, and Eisai. Elai Davicioni, James Proudfoot, and Yang Liu are employees of Veracyte, Inc. Other authors declare that they have no known competing financial interests or personal relationships that could have appeared to influence the work reported in this paper.

## Appendix Supplementary materials

Image, application 1
