# Supplementary material for: Transcriptomic predictors of prostate cancer recurrence following focal cryotherapy: a pooled analysis of phase II trial and prospective cohort data
Source: J Natl Cancer Cent. 2025 May 29;5(5):515–23. doi: 10.1016/j.jncc.2025.04.002 (PMC12529604; doi:10.1016/j.jncc.2025.04.002)
Supplement: Supplementary file 1 [file mmc1.pdf]

Supplementary materials

Transcriptomic predictors of prostate cancer recurrence following focal cryotherapy: a pooled analysis of phase II trial and prospective cohort data

Kae Jack Tay, Boon Hao Hong, Enya Hui Wen Ong, Kah Min Tan, Gianella Cabuhat Pacho, Samantha Jingxuan Wong, Yu Guang Tan, Yan Mee Law, Nye Thane Ngo, Puay Hoon Tan, John S.P. Yuen, Henry S.S. Ho, Kenneth Chen, Jiping Peng, Clare Wei Tian Foo, Xin Xiu Sam, Jeffrey K.L. Tuan, Ravindran Kanesvaran, Rajan T. Gupta, Steven Rozen, Thomas J. Polascik, Yang Liu, James Proudfoot, Elai Davicioni, Li Yan Khor, Melvin Lee Kiang Chua

Supplementary methods

Clinical trial and prospective observational cohort clinical protocol

Detailed ethics and consent

We included patients from a single-arm Phase II prospective clinical trial (NCT04138914/ ethics approval number: Singhealth IRB 2018/2482) enrolling patients with localized, untreated, non-metastatic clinically significant prostate cancer (csPCa) for focal therapy (FT) using focal cryotherapy (FCT) between October 2019 to April 2021 (Median age: 71.5 years, IQR: 64.8-74.2)[1]. From May 2021 onwards, patients were enrolled using the same trial inclusion criteria and follow-up protocol in a prospective observational (PO) cohort (median age:65.9 years, IQR: 61.2-71.1) extension of the clinical trial (ethics approval number: Singhealth IRB 2018/2014). Informed consent was also obtained from all patients to use their biopsy specimens for genomic sequencing (ethics approval number: Singhealth IRB 2020/2845).

The sole difference between the clinical trial and the PO cohort was that the focal cryotherapy treatment was research grant-sponsored in the clinical trial, and patient-sponsored in the prospective observational cohort. The patient selection, inclusion, treatment, and follow-up protocols were otherwise identical and are detailed below.

Patient selection - MRI and biopsy

All patients underwent 3-Tesla mpMRI of the prostate (Magnetom Skyra, Siemens Healthineers, Erlangen, Germany). High resolution images were acquired with a 60-channel pelvic phase array

coil. When performed in our institution, mpMRI was performed using our PIRADSv2.1 compliant standard institutional protocol which was previously described[2]. Briefly, multiplanar small field of view T2W TSE performed in axial, sagittal and coronal planes (FOV 20 × 20 cm) and axial T1W (FOV 20 x 20 cm) were the anatomical sequences acquired. Axial high B-value diffusion weighted imaging (DWI) with b-values b-0, 500, 1000, 1800 s/mm2 (FOV 20 × 20 cm) and axial dynamic contrast enhanced imaging (DCE) temporal resolution 4.47 seconds (FOV 26 × 26 cm) following a bolus of gadolinium contrast agent (Gadovist, Schering AG, Germany) at dose of 0.1 mmol/kg were the functional sequences acquired. Apparent diffusion coefficient (ADC) mapping was acquired with all available B-values. Experienced uro-radiologists with 3 to 8 years' experience reading MRI prostate interpreted the MRI images using PIRADSv2.1 criteria. Each lesion was graded and assigned a lesion specific overall PIRADSv2.1 category. A maximum of 4 lesions were identified on the mpMRI.

All lesions assigned PIRADSv2.1 category ≥ 3 was marked for MRI-TRUS fusion targeted biopsy. All patients then underwent a robotic transperineal MRI-targeted followed by a systematic saturation prostate biopsy of at least 20 cores[3]. The mpMRI and biopsy outcomes were reviewed by a multidisciplinary team (MDT), comprising of a radiologist, pathologist and treating urologist for eligibility.

Two recent expert consensuses on patient selection for FT[4,5] were used to develop our inclusion criteria. We included patients with a PSA level < 20ng/ml, biopsy prognostic grade group (GG) ≤ 4, mpMRI demonstrating no extracapsular extension and mpMRI determined index lesion volume of ≤ 3ml for single lesions or ≤ 1.5 ml each for multiple lesions. We permitted patients to undergo FT of up to two clinically significant (≥ GG2) lesions at any one time. Patients who had undergone prior treatment for prostate cancer other than active surveillance were excluded. Baseline patient reported outcome measures were recorded using the Expanded Prostate Cancer Index Composite questionnaire (EPIC).

Surgical procedure

Our technique of focal cryotherapy has been previously described[5]. All patients underwent focal cryotherapy (Visual ICE™, Boston Scientific, Boston, USA) as a day procedure under general anesthesia. The number and type of cryoprobes were determined by the treating physician to achieve a 5 mm treatment margin of < 20 degrees Celsius around the target lesion/ zone[6]. The cryoprobes were inserted transperineally under transrectal ultrasound (TRUS) guidance using a biplanar ultrasound probe (BK Ultrasound, city, USA). Temperature probes were placed to monitor critical

structures (the urethral sphincter, the anterior rectal wall and/or the neurovascular bundle) and the treatment margin where needed. A urethral warmer was placed to protect the urethra. A total of two freeze-thaw cycles were performed with the extent of iceball formation monitored using real-time TRUS. Patients who were unable to void post-procedure were catheterized with a foley catheter for 2-5 days.

Oncological follow up

Follow-up comprised serum PSA testing at 1, 3, 6, 12 months after focal cryotherapy. At 12 months post-treatment, all patients underwent a mandatory repeat mpMRI followed by transperineal prostate re-biopsy, comprising of (1) targeted biopsy over the previous ablation sites, (2) targeted biopsy of PIRADSv2.1 category ≥ 3 lesions detected on follow up mpMRI (3) systematic saturation biopsy of the remaining prostate. PIRADSv2.1, while strictly designed for treatment naïve prostate gland, we believed it remains relevant for assessment of recurrent tumor post focal treatment.

FT preserves a significant amount of prostate tissue post ablation, we applied the widely established PIRADSv2.1 in assessment of residual prostate tissue post cryoablation in surveillance mpMRI. Patients with residual or recurrent clinically significant cancer were re-evaluated at our MDT for active surveillance (AS), repeat FCT or radical treatment. Patients without clinically significant cancer were monitored using our standard institution AS protocol.

Gene expression profiling

Gene expression profiling was licensed for research use at the National Cancer Centre Singapore (NCCS) following the protocols used for microarray processing in a CLIA-certified clinical operations laboratory (Veracyte Inc, CA). Gene expression data was based on the Human Exon 1.0 ST oligonucleotide microarray (ThermoFisher, CA) to measure the expression of 46,050 genes and non-coding RNA transcripts. Microarray data were normalized using Single Channel Array Normalization.

The Decipher genomic classifier (GC) score was reported as a continuous score of 0 to 1, with 1 representing the most unfavorable biology[7]. Briefly, GC score was computed based on locked random forest model of 22 genes to produce a score between 0 and 1[7]. The GC score was further classified into 3 risk-categories of low-, intermediate-, and high-risk based on established cut-offs of < 0.45, 0.45-0.60, and > 0.60[8].

Exploratory data analyses

Patient-level analysis

To assess the predictive ability of the GC score for 1-year post-FT csPCa recurrence, it was compared to GG and PIRADS category. For patients with multifocal PCa, the highest GC score, GG, and PIRADS category were used as representative data points. Patients from both cohorts were pooled for this analysis (n = 52). The GC risk group was further used to assess the thresholds defined previously. The odds ratios for age, PSA at diagnosis, focality of the patient (multifocal *vs* unifocal), MRI tumor size (largest dimension in cm), PIRADS category (PIRADS 5 *vs* PIRADS < 5), GG, NCCN risk group, and GC score were estimated using univariable logistic regression analysis. Given that the limitation number of event in this study (n = 9), multivariable logistic regression was utilized to adjust for the confounding effects between the GC score and GG only.

Lesion-level analysis

To explore the transcriptomic signatures for patients, all the lesions (n = 83) sampled from 52 patients were assessed in this analysis to increase the statistical power. To access the differences in transcriptomic signatures (basal-luminal status, tumor immune microenvironments, and cancer hallmark pathways) between the lesions related to csPCa recurrences (n = 12) and non-related to csPCa recurrence (n = 71), the required signatures data were downloaded from the Genomic Research Intelligent Discovery (GRID) database for each lesion. Prostate Subtyping Classifier (PSC)[9] and Prediction Analysis of Microarray 50 (PAM50)[10] were used to examine the association of the basal-luminal status of the lesions with csPCa recurrence lesions. The differences between 27 immune cell signatures[11] with csPCa recurrence related lesions were explored. Odd ratio was used to measure the effect of 38 cancer hallmark signatures[12] that selected in previous work[13] towards csPCa recurrence lesions in univariable logistic regression analysis. The basal-luminal subtypes and cancer hallmark signatures reached statistically significant ($P < 0.05$) were further adjusted the confounder effect with GG in multivariable logistic regression analysis.

Statistical test

The Mann-Whitney U and Kruskal-Wallis tests were used to compare categorical and continuous variables, while the Chi-squared test was used for comparisons between two categorical variables. Spearman's rank correlation test was employed to examine the correlation between selected variables (GC score *vs* GG, and GC score *vs* PIRADS category). PAM50 and PSC were analyzed as categorical variables based on established cut-off points[14], whereas those that did not have a 'locked' cut-point like immune cell signatures, cancer hallmark signature scores were analyzed as continuous

variables. Due to the low number of csPCa recurrence in this this study, Firth's bias-reduced logistic regression[15] was used for all logistic regression analyses (both univariable and multivariable) for both patient level and lesion level analyses. All categorical variables assessed in the logistic regression analysis were binned as necessary to ensure the model converged and provided reasonable odds ratio estimates. Given the exploratory nature of these analyses, multiple hypothesis testing was not performed in this study.

References

1. Tan YG, Law YM, Ngo NT, et al. Patient-reported functional outcomes and oncological control after primary focal cryotherapy for clinically significant prostate cancer: A Phase II mandatory biopsy-monitored study. *Prostate*. 2023;83(8):781-791. doi:10.1002/pros.24517
2. Aslim EJ, Law YXT, Fook-Chong SMC, et al. Defining prostate cancer size and treatment margin for focal therapy: does intralesional heterogeneity impact the performance of multiparametric MRI? *BJU Int*. 2021;128(2):178-186. doi:10.1111/bju.15355
3. Lee AYM, Chen K, Tan YG, et al. Reducing the number of systematic biopsy cores in the era of MRI targeted biopsy-implications on clinically-significant prostate cancer detection and relevance to focal therapy planning. *Prostate Cancer Prostatic Dis*. 2022;25(4):720-726. doi:10.1038/s41391-021-00485-3
4. Tay KJ, Scheltema MJ, Ahmed HU, et al. Patient selection for prostate focal therapy in the era of active surveillance: an International Delphi Consensus Project. *Prostate Cancer Prostatic Dis*. 2017;20(3):294-299. doi:10.1038/pcan.2017.8
5. Scheltema MJ, Tay KJ, Postema AW, et al. Utilization of multiparametric prostate magnetic resonance imaging in clinical practice and focal therapy: report from a Delphi consensus project. *World J Urol*. 2017;35(5):695-701. doi:10.1007/s00345-016-1932-1
6. Tan YG, Yuen JS, Ho HS, et al. Key steps in the evaluation and treatment planning for prostate focal cryotherapy. *Videourology*. 2021;35(8).
7. Erho N, Crisan A, Vergara IA, et al. Discovery and validation of a prostate cancer genomic classifier that predicts early metastasis following radical prostatectomy. *PLoS One*. 2013;8(6):e66855. doi:10.1371/journal.pone.0066855

8. Ross AE, Johnson MH, Yousefi K, et al. Tissue-based Genomics Augments Post-prostatectomy Risk Stratification in a Natural History Cohort of Intermediate- and High-Risk Men. *Eur Urol*. 2016;69(1):157-165. doi:10.1016/j.eururo.2015.05.042

9. Weiner AB, Liu Y, Hakansson A, et al. A novel prostate cancer subtyping classifier based on luminal and basal phenotypes. *Cancer*. 2023;129(14):2169-2178. doi:10.1002/cncr.34790

10. Zhao SG, Chang SL, Erho N, et al. Associations of Luminal and Basal Subtyping of Prostate Cancer With Prognosis and Response to Androgen Deprivation Therapy. *JAMA Oncol*. 2017;3(12):1663-1672. doi:10.1001/jamaoncol.2017.0751

11. Charoentong P, Finotello F, Angelova M, et al. Pan-cancer Immunogenomic Analyses Reveal Genotype-Immunophenotype Relationships and Predictors of Response to Checkpoint Blockade. *Cell Rep*. 2017;18(1):248-262. doi:10.1016/j.celrep.2016.12.019

12. Liberzon A, Birger C, Thorvaldsdóttir H, Ghandi M, Mesirov JP, Tamayo P. The Molecular Signatures Database (MSigDB) hallmark gene set collection. *Cell Syst*. 2015;1(6):417-425. doi:10.1016/j.cels.2015.12.004

13. Chua MLK, Hakansson AK, Ong EHW, et al. Transcriptomic analyses of localized prostate cancers of East Asian and North American men reveal race-specific luminal-basal and microenvironmental differences. *Cancer Commun (Lond)*. 2023;43(10):1164-1168. doi:10.1002/cac2.12467

14. Parry MA, Grist E, Mendes L, et al. Clinical testing of transcriptome-wide expression profiles in high-risk localized and metastatic prostate cancer starting androgen deprivation therapy: an ancillary study of the STAMPEDE abiraterone Phase 3 trial. *Res Sq [Preprint]. 2023 Feb 8:rs.3.rs-2488586.* doi:10.21203/rs.3.rs-2488586/v1

15. Puhr R, Heinze G, Nold M, Lusa L, Geroldinger A. Firth's logistic regression with rare events: accurate effect estimates and predictions? *Stat Med*. 2017;36(14):2302-2317. doi:10.1002/sim.7273

Supplementary Table 1

Summary information for patients with discordant highest GC scores and highest GG.

Patient ID	Age, years	PSA	GG	GC	PIRADS
P06	47.7	11.0			
Lesion 1*			3	0.62	4
Lesion 2			1	0.63	4
Lesion 3			1	0.43	3
P17					
Lesion 1*	75.8	3.6	3	0.52	4
Lesion 2			2	0.56	MRI invisible
Lesion 3			1	0.40	3

* Clinical index lesion. The Patient ID referred to the x-axis in Fig. 2A.

Abbreviations: GC, genomic classifier; GG, Gleason grade; MRI, magnetic resonance imaging; PIRADS, Prostate-Imaging Reporting and Data System; PSA, prostate-specific antigen.

Supplementary Table 2

Multivariable logistics regression analysis between clinically significant prostate cancer recurrence and GC score by adjusted with PIRADS.

	Multivariable*	
	Odd ratio (95% CI)	*P*
PIRADS		
< 5	Reference	
5	0.20 (0.01-1.30)	0.097
GC score	1.56 (1.13-2.37)	0.006

* The number of patients include: csPCa recurrence, yes (n = 9); no (n = 43).

Abbreviations: CI, confidence interval; GC, genomic classifier; PIRADS, Prostate-Imaging Reporting and Data System.

Supplementary Table 3

Multivariable logistics regression analyses of clinically significant prostate cancer recurrence related lesions with PSC subtype and adjusted by grade group of each lesion.*

	Univariable		Multivariable	
	Odd ratio (95% CI)	*P*	Odd ratio (95% CI)	*P*
PSC				
LP/BI/BN	Reference		Reference	
LD	0.07 (0.00-0.56)	0.007	0.09 (0.00-0.69)	0.015
Grade group				
1	Reference		Reference	
2	2.72 (0.54-27.03)	0.244	1.85 (0.34-19.01)	0.502
3	5.28 (0.75-60.25)	0.096	3.79 (0.49-45.64)	0.204
4	8.60 (0.55-147.10)	0.117	7.26 (0.40-164.34)	0.172

* The number of patients include: csPCa recurrence, yes (n = 12); no (n = 71).

Abbreviations: CI, confidence interval; BI, basal-immune; BN, basal-neuroendocrine; LD, luminal-differentiated; LP, luminal-proliferative; PSC, Prostate-specific subtyping classifier.

Supplementary Table 4

Multivariable logistics regression analyses of clinically significant prostate cancer recurrence related lesions with DNA repair hallmark signature scores and adjusted by grade group of each lesion.

	Multivariable*	
	Odd ratio (95% CI)	*P*
DNA repair	1.94 (0.97-4.20)	0.061
Grade group		
1	Reference	
2	2.11 (0.40-21.26)	0.404
3	2.75 (0.34-33.41)	0.345
4	9.42 (0.52-189.70)	0.119

* The number of patients include: csPCa recurrence, yes (*n* = 12); no (*n* = 71).

Abbreviations: CI, confidence interval.
